# The Influence of Negative Emotion on Cognitive and Emotional Control Remains Intact in Aging

**DOI:** 10.3389/fnagi.2017.00349

**Published:** 2017-11-01

**Authors:** Artyom Zinchenko, Christian Obermeier, Philipp Kanske, Erich Schröger, Arno Villringer, Sonja A. Kotz

**Affiliations:** ^1^International Max Planck Research School on Neuroscience of Communication, Leipzig, Germany; ^2^Department of Neuropsychology, Max Planck Institute for Human Cognitive and Brain Sciences, Leipzig, Germany; ^3^Department Psychologie, Ludwig-Maximilians-Universität München, Munich, Germany; ^4^Department of Social Neuroscience, Max Planck Institute for Human Cognitive and Brain Sciences, Leipzig, Germany; ^5^Institute of Clinical Psychology and Psychotherapy, Department of Psychology, Technische Universität Dresden, Dresden, Germany; ^6^Institute of Psychology, University of Leipzig, Leipzig, Germany; ^7^Department of Neuropsychology and Psychopharmacology, Faculty of Psychology and Neuroscience, Maastricht University, Maastricht, Netherlands

**Keywords:** aging, executive control, conflict processing, cognitive conflict, emotional conflict, ERPs

## Abstract

Healthy aging is characterized by a gradual decline in cognitive control and inhibition of interferences, while emotional control is either preserved or facilitated. Emotional control regulates the processing of emotional conflicts such as in irony in speech, and cognitive control resolves conflict between non-affective tendencies. While negative emotion can trigger control processes and speed up resolution of both cognitive and emotional conflicts, we know little about how aging affects the interaction of emotion and control. In two EEG experiments, we compared the influence of negative emotion on cognitive and emotional conflict processing in groups of younger adults (mean age = 25.2 years) and older adults (69.4 years). Participants viewed short video clips and either categorized spoken vowels (cognitive conflict) or their emotional valence (emotional conflict), while the visual facial information was congruent or incongruent. Results show that negative emotion modulates both cognitive and emotional conflict processing in younger and older adults as indicated in reduced response times and/or enhanced event-related potentials (ERPs). In emotional conflict processing, we observed a valence-specific N100 ERP component in both age groups. In cognitive conflict processing, we observed an interaction of emotion by congruence in the N100 responses in both age groups, and a main effect of congruence in the P200 and N200. Thus, the influence of emotion on conflict processing remains intact in aging, despite a marked decline in cognitive control. Older adults may prioritize emotional wellbeing and preserve the role of emotion in cognitive and emotional control.

## Introduction

In everyday social interactions, we observe multiple non-verbal cues such as facial expressions, gestures, and vocalizations (e.g., during an informal talk with a colleague, in a job interview). For instance, simple vocalizations can describe people’s emotions, feelings, and even social attitudes (Mohammadi and Vinciarelli, 2011). Therefore, the ability to accurately perceive emotional vocalizations is an important aspect of social communication and hampered recognition of emotional states may lead to inappropriate social behavior and poor interpersonal communication (Shimokawa et al., 2001; Blair, 2005; Chaby et al., 2015). However, this task becomes even more challenging when there is conflicting emotional information, for instance, when the affective meaning of vocal and facial expressions are incongruent (e.g., in irony; Wang et al., 2007; Pexman, 2008; Watanabe et al., 2014; Zinchenko et al., 2015). Such conflict usually results in prolonged reaction times (RTs) and increased error rates (Stroop, 1935; Simon and Rudell, 1967; Egner and Hirsch, 2005; Ochsner et al., 2009) reflecting increased demand on limited attentional resources when processing incongruent information (West, 2003).

We recently showed that negative emotion can facilitate emotional conflict processing (Zinchenko et al., 2015) similarly to its influence on cognitive conflict processing (e.g., Stroop task) where a conflict arises between opposing non-emotional stimulus dimensions (Kanske and Kotz, 2010, 2011a,b). Negative emotions lead to faster processing of both cognitive and emotional conflicts, and to a conflict-specific event-related potentials (ERPs; Zinchenko et al., 2015).

More specifically, our previous study showed a conflict specific dissociation for the N100 (Zinchenko et al., 2015). The N100 is a negative deflection occurring approximately 100 ms after stimulus onset. The N100 is modulated by attention (Hillyard et al., 1973), emotion (Scott et al., 2009), and congruence (Pourtois et al., 2000; Atkinson et al., 2003). We reported a larger conflict effect for emotional than for neutral trials in the cognitive task and a larger conflict effect for neutral than for emotional trials in the emotional task (Zinchenko et al., 2015). Furthermore, the P200 (positive-going deflection approximately 200 ms after stimulus onset) has been shown to increase for emotional stimuli (Paulmann and Kotz, 2008; Kanske et al., 2011), while processing of incongruity leads to a decreased P200 amplitude (Kokinous et al., 2014). Additionally, previous findings showed a reduced P200 response to incongruent compared to congruent trials in both cognitive and emotional tasks (Kokinous et al., 2014; Zinchenko et al., 2015). Finally, the N200 has been repeatedly reported as a neural marker of cognitive control and conflict processing with a larger N200 amplitude in response to incongruent than congruent stimuli (Kopp et al., 1996a; Van Veen and Carter, 2002; Luck, 2005; Azizian et al., 2006). This effect has been found at fronto-central (Kopp et al., 1996b; Heil et al., 2000; Kanske and Kotz, 2011a) as well as at posterior electrode-sites (Hughes et al., 2009; Zinchenko et al., 2015).

However, we know little about age-related changes in how emotion affects the two types of conflict. As people get older their ability to process cognitive conflict declines (Hasher et al., 1991). Studies reported that older adults show larger conflict effects and exhibit consistently poorer performance on tasks that require inhibition of interference (Stoltzfus et al., 1993). Importantly, this increased conflict effect in the elderly is not the result of general slowing, but is thought to reflect age-related decline in executive control and inhibition of interference (Langenecker et al., 2004; Zysset et al., 2007). On the other hand, inhibition of *affective* interference seems improved in older adults. For instance, Samanez-Larkin et al. (2009) showed that older but not younger adults show no emotional conflict cost when the task is to judge the emotional valence of a target word flanked by congruent or incongruent emotional distractors (i.e., negative, positive). Nonetheless, both age groups showed interference on control trials that required processing of non-emotional targets and distractors (Samanez-Larkin et al., 2009). Importantly, the results showed that older adults had no difficulties in identifying the emotional valence of stimuli in general. These findings are consistent with the growing body of literature suggesting that the ability to process and regulate emotions effectively remains stable or even improves across the adult lifespan (Charles and Carstensen, 2007). According to the socio-emotional selectivity theory, older adults prioritize processing and regulation of emotional information; their goals increasingly emphasize emotional components of life (Carstensen, 1993, 2006; Carstensen et al., 1999). Thus, older adults can better overcome negative emotions and thoughts (Carstensen et al., 2000), regulate emotions and display less physiological arousal when experiencing negative emotions (Tsai et al., 2000; Samanez-Larkin and Carstensen, 2011) than younger adults.

Conversely, some studies showed that older adults display decreased accuracy in emotion recognition portrayed in facial expressions (pictures of emotional faces; e.g., Williams et al., 2009; Orgeta, 2010; Lambrecht et al., 2012), conveyed through prosodic cues (Kiss and Ennis, 2001; Paulmann et al., 2009; Mitchell et al., 2011; Lambrecht et al., 2012; Templier et al., 2015) and, particularly, through non-verbal vocal cues (e.g., screams or laughter; Hunter et al., 2010; Lima et al., 2014; Chaby et al., 2015).

Thus, there is mixed evidence regarding the processing of emotions in older adults. Most importantly, it is not clear whether and how the emotional quality of a target (emotional, neutral) influences cognitive and emotional conflict processing as a function of age. Moreover, when using an emotional conflict task, previous studies employed stimuli where both the target and non-targets were emotional and there was no neutral baseline control (Egner et al., 2008; Samanez-Larkin et al., 2009). Therefore, the present study tested whether in older adults negative compared to neutral stimuli influence cognitive and emotional conflict processing differentially.

We used an experimental protocol that varied the source of conflict between non-emotional (cognitive conflict) and emotional (emotional conflict) stimulus dimensions while using identical stimuli in the exploration of both conflict types. We also measured ERPs to study neural correlates of early conflict-specific perceptual processes (e.g., N100; Zinchenko et al., 2015) and the influence of emotion on the two types of conflict (N200; Bruin and Wijers, 2002; Kanske and Kotz, 2011a). In two ERP studies, participants saw multisensory dynamic stimuli: short video clips of actors pronouncing different vocalizations in a negative or neutral tone of voice. Auditory and visual coding of the stimuli could be either congruent or incongruent with respect to vocalization (cognitive conflict task, Experiment 1) or emotion (emotional conflict task, Experiment 2). In Experiment 1, participants named the vowel expressed by the voice (i.e., “A” or “O”) irrespective of its emotional quality (emotional or neutral). In Experiment 2, participants were asked to report whether the voice was emotional or neutral, irrespective of the emotional valence of the face (i.e., the source of conflict was emotional). Importantly, while stimuli were identical in both tasks, the source of conflict was either emotional or cognitive and the target dimension was either emotional or neutral. This allowed testing whether the affective quality of the target (emotional, neutral) influences executive control when it is task-irrelevant (cognitive conflict, Experiment 1) and task-relevant (emotional conflict, Experiment 2).

Emotional and cognitive information processed via multisensory channels optimizes behavioral responses in both younger (de Gelder and Vroomen, 2000; Kreifelts et al., 2007; Klasen et al., 2011) and older adults (Laurienti et al., 2006; Peiffer et al., 2007; Diederich et al., 2008; Hugenschmidt et al., 2009; DeLoss et al., 2013). Therefore, we used dynamic multisensory videos in order to achieve optimal performance (e.g., Jessen and Kotz, 2011; Klasen et al., 2012; Donohue et al., 2013). Based on previous findings and the aging literature, we hypothesized that older compared to younger adults may be *more* susceptible to interference in the *cognitive conflict* task (Langenecker et al., 2004; Zysset et al., 2007) and be *less* susceptible to affective interference in the *emotional conflict* task (Samanez-Larkin et al., 2009). We also expected that emotional targets would facilitate both types of conflict processing in younger adults (Kanske and Kotz, 2011a; Zinchenko et al., 2015). Whether or not we would observe similar facilitation in older adults was less clear as this has not yet been investigated. However, as several previous studies indicated preserved capabilities to process emotions in older participants (Mikels et al., 2005; Charles and Carstensen, 2007) and showed that both age groups use similar neural mechanisms to process emotions (Ebner et al., 2012), we expected that emotional targets would facilitate both types of conflicts in older adults as well.

While we found a larger conflict effect for emotional compared to neutral trials in the *cognitive* task and a larger conflict effect for neutral compared to emotional trials in the *emotional* task (Zinchenko et al., 2015), a comparable study on cognitive and emotional conflict processing did not observe a similar N1 dissociation (Alguacil et al., 2013). On the other hand, in line with previous findings, we expected to observe a reduced P200 response to incongruent than congruent trials in both cognitive and emotional tasks (Kokinous et al., 2014; Zinchenko et al., 2015). Furthermore, we expected to observe conflict-related N100 and P200 components either over anterior (Liu et al., 2012; Ho et al., 2015; Kokinous et al., 2015) or posterior electrode-sites (Gerdes et al., 2013; Ho et al., 2015).

Finally, we expected to observe larger N200 responses for incongruent compared to congruent stimuli in both age groups across the scalp (Zinchenko et al., 2015; Korsch et al., 2016). Since the modulation of the conflict-sensitive N200 response has been reported for unimodal stimuli (Kanske and Kotz, 2011b), but not for multisensory stimuli (Zinchenko et al., 2015), the question remains whether we would observe a larger N200 conflict effect for negative compared to neutral stimuli in the two conflict types and age groups.

## Experiment 1

### Methods

#### Participants

Twenty-six younger adults (female = 14, mean age = 25.2 years, range = 21–35) and 26 older adults (female = 13, mean age = 69.4 years, range = 63–78), all right-handed (Edinburgh Handedness Inventory score ME = 88.6, SD = 12.1) with normal or corrected-to-normal vision and self-reported normal hearing participated in the two experiments. Participants underwent medical screening for any neurological history and were screened for past and present psychiatric disorders using the Structured Clinical Interview in DSM-IV (SCID-I; Wittchen et al., 1995) at the Day Clinic for Cognitive Neurology, University of Leipzig. None of participants had a history of psychiatric/neurological disorders, alcoholism, or drug abuse. Drug abuse was further controlled in both age groups by an instant dipstick drug test (Drogentest Multi-8/2-DT, Diagnostik Nord) that examined the use of eight drugs (amphetamine, buprenorphine, benzodiazepines, cocaine, methamphetamine, morphine/opiates, methadone, and cannabis). Younger and older adults differed in mean years of education: younger adults (all 12 years), older adults [mean = 10.9 years, SD = 1.56, *t*(22) = 3.27, *p* < 0.01]. Both age groups were recruited from the database of the “Leipzig Cohort for Mind-Body-Emotion Interactions” (LEMON). Older adults completed a pure-tone audiometric screening: participants were included only if they had thresholds equal to or lower than 30 dB in both ears at frequencies crucial for speech perception (500–4000 Hz, Lima et al., 2014). Participants filled out the Adult Temperament Questionnaire (effortful and attentional control subscales, ATQ; Derryberry and Rothbart, 1988) and Depression Anxiety Stress Scale (DASS; Lovibond and Lovibond, 1995). Older adults showed lower levels of inhibitory control (4.5) relative to younger adults [5.29; *t*(50) = -4.49, *p* < 0.0001], lower levels of activation control [younger = 4.5, older = 5.58; *t*(50) = -3.84, *p* < 0.0001], lower attentional control: attentional shifting [younger = 4.7, older = 5.51; *t*(50) = -2.8, *p* < 0.01], and effortful control [younger = 4, older = 4.6; *t*(50) = -3.07, *p* < 0.01]. We also observed that older adults displayed higher levels of stress (13.31) than younger adults [7.76; *t*(50) = -3.39, *p* < 0.001], as well as higher levels of anxiety [younger = 2.3, older = 8.4; *t*(50) = -5.71, *p* < 0.0001] and depression [younger = 4.46, older = 9.85; *t*(50) = -3.17, *p* < 0.01]. Participants also rated the complete videos, video streams alone, and audio streams alone on a 7-point Likert scale using Self-Assessment Manikins for expressiveness, arousal, and *emotion identification* (Bradley and Lang, 1994; see **Table 1**). In order to control for the correct emotion identification in the younger and older adults, we used the ratings of the neutral stimuli by the younger adults from a previous study (Zinchenko et al., 2015) to extract the upper and lower boundaries of the confidence interval. If the neutral videos’ ratings of participants in the current study fell within the boundaries of the confidence interval, and the ratings of emotional videos fell outside of these boundaries, the participant identified emotion expressed in the stimuli correctly. This procedure ensured that participants were able to identify emotional stimuli, as the task required processing of emotions (three participants were not able to identify emotion of voices and were not included in further tests). Both groups showed no differences at the level of perceived expressiveness and arousal of the stimuli and rated emotional material as more emotional compared to neutral material (see Supplementary Material for details). All participants signed a consent form and received payment for their participation. The experiment followed the guidelines of the Declaration of Helsinki and was approved by the Ethics Committee of the University of Leipzig.

**Table 1:** Results of the video rating.

Stimuli		Arousal	Expressiveness	Valence
**Younger adults**				
Complete video	Neutral	5.18 (3.21)	5.41 (2.78)	4.95 (0.24)
	Negative	4.62 (2.40)	4.63 (2.86)	1.71 (0.55)
Audio stream	Neutral	4.63 (1.32)	4.44 (1.43)	4.94 (0.24)
	Negative	5.10 (1.26)	4.94 (1.28)	1.74 (0.55)
Videos stream	Neutral	5.36 (2.98)	5.31 (3.07)	4.98 (0.25)
	Negative	5.02 (2.19)	4.82 (2.56)	1.88 (1.32)
**Older adults**				
Complete video	Neutral	5.13 (2.84)	5.35 (2.14)	4.97 (0.23)
	Negative	4.94 (2.71)	4.76 (2.23)	1.73 (0.52)
Audio stream	Neutral	4.72 (1.13)	4.12 (1.08)	4.97 (0.27)
	Negative	4.81 (0.98)	4.43 (1.05)	1.24 (0.53)
Videos stream	Neutral	5.05 (1.43)	4.63 (1.46)	4.97 (0.25)
	Negative	4.88 (1.36)	4.68 (0.88)	2.57 (1.33)

#### Stimulus Material

**FIGURE 1:**
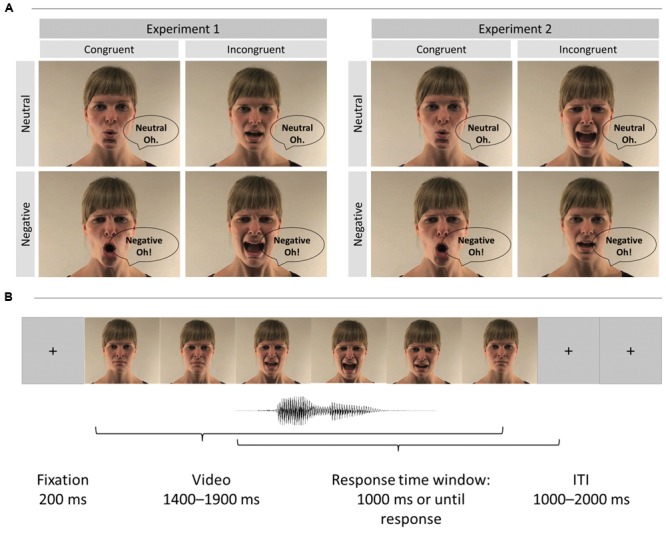
**(A)** Examples of video stimuli in Experiments 1 and 2: the female actor (the actor signed a consent form for the publication of these images) vocalized the interjections “A” and “O” in either a negative or a neutral tone of voice. Incongruence was created by mismatches in the vocalizations of audio and video components in Experiment 1, and mismatches in the expressed emotion of audio and video components in Experiment 2. **(B)** Example of a trial sequence.

Stimuli, design, and procedure in the current study were identical to and validated in our previous work (Zinchenko et al., 2015). Stimuli consisted of short video clips of a male (27 years old) and a female (24 years old) semi-professional actors pronouncing the interjections “A” and “O” in a neutral and negative (i.e., angry) tone of voice (see **Figure 1A**). The sound in all videos was normalized to 70 dB using root mean square in Final Cut Pro. We created eight congruent and eight incongruent videos by matching or mismatching vocalizations of the face and voice (e.g., voice pronouncing “A” with facial lip movement corresponding to “A” vs. “O,” respectively). Videos were overlaid with the incongruent sound in Final Cut Pro 7 (Apple Inc.) using the onset of the original video sound for alignment. The duration of congruent and incongruent videos ranged between 1 and 2 s (**Table 2**). There were no time differences between conditions before the audio onset and the total video durations (see Supplementary Material for details).

**Table 2:** Timing of video stimuli of Experiment 1.

Video condition (the “vowel” specifies the interjection)	Time before start of the movement (ms)	Time before start of the audio sound (ms)	Total video duration (ms)
**Female**			
Neutralcongruent “A”	240	561	1400
Neutral congruent “O”	240	740	1480
Negative congruent “A”	240	665	1880
Negative congruent “O”	240	846	1840
Face neutral “A”–voice neutral “O”	240	540	1400
Face neutral “O”–voice neutral “A”	240	562	1480
Face negative “A”–voice negative “O”	240	680	1880
Face negative “O”–voice negative “A”	240	630	1840
**Male**			
Neutral congruent “A”	240	475	1400
Neutral congruent “O”	240	560	1400
Negative congruent “A”	240	450	1400
Negative congruent “O”	240	540	1400
Face neutral “A”–voice neutral “O”	240	520	1400
Face neutral “O”–voice neutral “A”	240	635	1400
Face negative “A”–voice negative “O”	240	580	1400
Face negative “O”–voice negative “A”	240	490	1400

To check whether videos differed with regards to movement, we estimated the number of movements per video clip by quantifying the variation of light intensity (luminance) between pairs of frames for each pixel (Pichon et al., 2008). The two emotions (neutral and emotional) and two vowels (“A” and “O”) were compared with a Kruskal–Wallis test. Emotional videos showed a higher number of movements relative to neutral videos (χ^2^ = 5.33, *p* < 0.05). As it is an inherent feature of angry expressions to be more dynamic and intense (e.g., Weyers et al., 2006) differences in mean motion were expected in naturalistic stimuli. Most importantly, differences in movement should not affect the outcome of the results as we focused on the *interaction* of congruence and emotion. Motion differences were not significant for the different vowels (χ^2^ = 1.25, *p* > 0.2).

Experiment 1 used a 2 (emotional, neutral) × 2 (congruent, incongruent) factorial design and was split into four blocks. Each block comprised 52 videos (26 emotional and 26 neutral, half were congruent and the other half incongruent) presented in a pseudo-randomized order. Overall, there were 208 trials and testing took approximately 45 min per participant.

#### Procedure

Participants sat in a dimly lit sound-attenuated booth about 1 m from a computer screen. The sound was delivered via headphones. Each trial started with a fixation cross on a blank computer screen for 200 ms (see **Figure 1B**). Subsequently, a video was presented and played in full duration (i.e., video was not cut off after the response). Participants were instructed to report whether the vocalization of the voice was “A” or “O” irrespective of the emotional quality. The emotion of the face and voice was always matched. To ensure that faces were not ignored, participants were sometimes (10% of all trials) asked a second question about the vocalization (lip movement, i.e., “A” or “O”) of the face. These additional questions were presented randomly, the response time to these questions was not limited, and these trials were not used for further analysis (all participants answered >90% questions correctly and were included for further analysis). The response time-window to the main question was 1500 ms, starting from voice onset. If participants did not respond within the response time-window, the words “try to respond faster” appeared on the screen for 200 ms. If participants answered incorrectly the word “incorrect” appeared on the screen. Button presses were counterbalanced across participants. An ITI of 1000, 1250, 1500, 1750, and 2000 ms was used randomly before the onset of the next trial.

#### EEG Recording and Pre-processing

EEG was recorded from 59 Ag/AgCl scalp electrodes (10–10 system). We used Brain Vision Recorder (Brain Products GmbH, Munich, Germany) and a BRAINAMP amplifier (DC to 250 Hz). The sampling rate was 500 Hz. The left mastoid served as a reference, and the sternum as ground. The vertical and horizontal electro-oculogram was measured for artifact rejection purposes. The impedance was kept below 5 kΩ.

EEG data were analyzed using the FieldTrip (v0.20120501) toolbox (Oostenveld et al., 2011) running on Matlab 8.1 R2013a (The Mathworks, Inc., Natick, United States). Electrodes were re-referenced offline to linked mastoids. Only correct trials were chosen for data processing. Extended epochs of 2000 ms before and after video onset were extracted. Epochs containing excessive muscle activity or jump artifacts were rejected. Data were band-pass filtered using a two-pass Butterworth IIR filter with a frequency pass-band of 0.1–100 Hz (order of 4). After the preprocessing, principal components analysis was applied in order to reduce dimensionality of the data and preserve α = 0.99 of the variance (Dien, 2012). An independent component analysis (ICA) was conducted using the *fastica* algorithm. Subsequently, components were visually inspected and those showing ocular, muscle, heart, and electrode artifacts were manually rejected (mean number of components removed = 11, SD = 4). In a subsequent step, individual epochs were visually scanned and epochs containing artifacts were manually discarded. Approximately 14% of trials (incorrect responses, artifacts) were excluded from further analysis.

#### Data Analysis

For the statistical analysis, smaller epochs were selected (-200 to 1000 ms time-locked to the voice onset). Single-subject averages were calculated for each session and condition. In accordance with previous literature (Kanske and Kotz, 2011a; Ho et al., 2015; Zinchenko et al., 2015), four regions of interest were defined: left anterior (FP1, AF3, AF7, F3, F5, F7, FC3, FC5, FT7), right anterior (FP2, AF4, AF8, F4, F6, F8, FC4, FC6, FT8), left posterior (CP3, CP5, TP7, P3, P5, P7, PO3, PO7, O1), and right posterior (CP4, CP6, TP8, P4, P6, P8, PO4, PO8, O2). Peak latencies were detected separately for each participant and each condition within the following time windows: 70–110 ms (N100), 140–225 ms (P200) as suggested by Luck and Kappenman (2011). The time window for the N200 was manually selected after visual inspection of the ERPs: 240–340 ms for younger adults and 280–380 ms for older adults. This was done due to the delayed latency of the N200 in older adults (Enoki et al., 1993; Anderer et al., 1996; Luck and Kappenman, 2011). A 20 ms time window was applied before and after each of the individual peaks for a mean amplitude analysis. For each time window a repeated-measures ANOVA was calculated, using emotion (emotional, neutral), congruence (congruent, incongruent), region (anterior, posterior) and side (left, right) as within-subject factors and age (younger, older) as a between-subject factor. As videos prior to voice onset in Experiment 1 were identical (as emotion of face and voice was kept constant and only vocalization varied) no baseline correction was applied before the voice onset (Zinchenko et al., 2015, 2017). Only statistically significant main effects and interactions that involved the critical factors emotion, congruence, and group are reported in the section “Results.”

Finally, as previous findings found a correlation between participants’ interpersonal characteristics (e.g., level of effortful control, anxiety, etc.) and the influence of emotion on executive attentional control (Kanske and Kotz, 2012) we have also ran an exploratory correlation analysis between the emotion-modulated N100 conflict effect [(negative incongruent - negative congruent) - (neutral incongruent - neutral congruent)] and the questionnaires data (i.e., ATQ, DASS).

### Results

#### Behavioral Data

##### RT data

**FIGURE 2:**
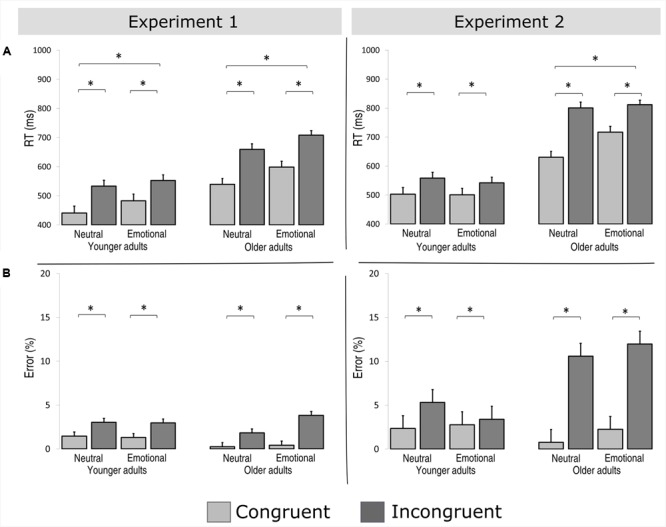
Reaction times **(A)** and error rates **(B)** data [mean + standard error of the mean (SEM)] for younger and older adults in the congruent and incongruent/emotional and neutral conditions of Experiment 1 (left) and Experiment 2 (right). ^∗^Denotes a significant main effect or interaction at *p* < 0.05.

The main effect of age was significant as older adults produced overall longer RTs than younger adults [see **Figure 2** and **Table 3**; younger = 502 ms, SD = 108 ms; older = 626 ms, SD = 96 ms; *F*(1,50) = 21.528, *p* < 0.001, η_p_^2^ = 0.301]. Emotional stimuli resulted in longer RTs than neutral stimuli [emotional = 585 ms, SD = 129 ms; neutral = 543 ms, 110 ms; *F*(1,50) = 66.27, *p* < 0.01, η_p_^2^ = 0.570]. Furthermore, we observed an interaction of emotion by age [*F*(1,50) = 10.92, *p* < 0.01, η_p_^2^ = 0.192]: the main effect of emotion was larger for older adults [54 ms, SD = 11 ms; *F*(1,25) = 41.89, *p* < 0.01, η_p_^2^ = 0.626] than for younger adults [30 ms; SD = 17 ms; *F*(1,25) = 24.39, *p* < 0.01, η_p_^2^ = 0.494]. Similarly, the RTs were prolonged for incongruent compared to congruent stimuli [congruent = 515 ms, SD = 111 ms; incongruent = 630 ms, SD = 135 ms; *F*(1,50) = 141.23, *p* < 0.01, η_p_^2^ = 0.739]. An interaction of congruence by age was also significant [*F*(1,50) = 141.23, *p* < 0.01, η_p_^2^ = 0.739] and confirmed that the conflict effect was larger for older [114 ms, SD = 11 ms; *F*(1,25) = 67.47, *p* < 0.01, η_p_^2^ = 0.730] than for younger adults [81 ms, SD = 4 ms; *F*(1,25) = 85.99, *p* < 0.01, η_p_^2^ = 0.775].

**Table 3:** Behavioral RT results of Experiments 1 and 2 per age group.

		Experiment 1		Experiment 2	
		Younger	Older	Younger	Older
Negative	Congruent	483.2	598.9	500.8	717.1
	Incongruent	552.7	708.3	542.5	811.8
Neutral	Congruent	440.9	539.4	502.9	630.3
	Incongruent	533.6	659.3	558.6	801.2

Finally, congruence was modulated by emotion [*F*(1,50) = 5.92, *p* < 0.02, η_p_^2^ = 0.106]: the conflict effect was smaller for negative [89 ms, SD = 2 ms; *F*(1,50) = 94.137, *p* < 0.01, η_p_^2^ = 0.653] than for neutral stimuli [106 ms, SD = 13 ms; *F*(1,50) = 151.58, *p* < 0.01, η_p_^2^ = 0.752]. This effect was comparable in both age groups as the interaction of emotion by congruence by age was not significant [younger = 23 ms, SD = 1 ms; older = 10 ms, SD = 20 ms; *F*(1,50) = 0.87, *p* > 0.3, η_p_^2^ = 0.017].

##### Error rate

Participants made more errors in response to incongruent (2.9%) than to congruent (0.86%) trials [*F*(1,50) = 5.06, *p* < 0.03, η_p_^2^ = 0.108]. No other effects reached a level of significance (all *p* > 0.1).

#### ERP Data

##### N100 range

**FIGURE 3:**
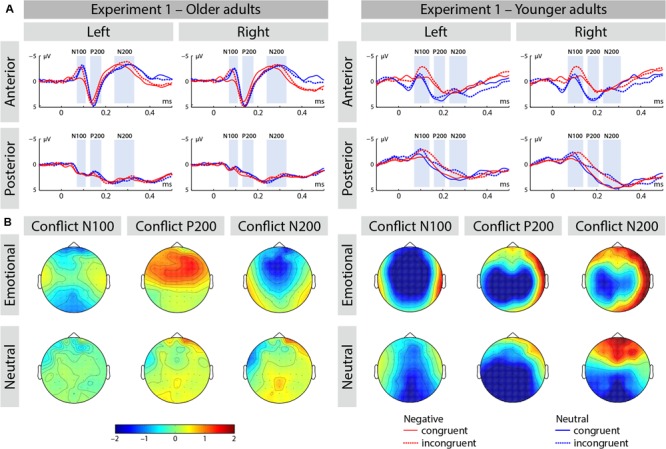
**(A)** ERP waveforms at selected averaged electrodes [left anterior (FP1, AF3, AF7, F3, F5, F7, FC3, FC5, FT7), right anterior (FP2, AF4, AF8, F4, F6, F8, FC4, FC6, FT8), left posterior (CP3, CP5, TP7, P3, P5, P7, PO3, PO7, O1), and right posterior (CP4, CP6, TP8, P4, P6, P8, PO4, PO8, O2)] depicting the conflict effect for emotional and neutral trials in Experiment 1. **(B)** Conflict effect represented as a topographic distribution of amplitude differences (incongruent–congruent) for each of the ERP components (i.e., N100, P200, and N200).

The main effect of congruence was significant [**Figure 3**; *F*(1,50) = 28.75, *p* < 0.001, η_p_^2^ = 0.365], as well as an interaction of congruence by emotion [*F*(1,50) = 9.32, *p* < 0.01, η_p_^2^ = 0.166]. As hypothesized, the conflict effect was larger in negative emotion stimuli [*F*(1,50) = 33.9, *p* < 0.001, η_p_^2^ = 0.399] relative to neutral stimuli [*F*(1,50) = 3.64, *p* > 0.05, η_p_^2^ = 0.067] and this effect was comparable across the both groups, as an interaction of emotion by congruence by age was not significant [*F*(1,50) = 0.101, *p* > 0.7, η_p_^2^ = 0.002]. Additionally, we found an interaction of emotion by age [*F*(1,50) = 23.99, *p* < 0.001, η_p_^2^ = 0.324]. Follow-up analysis revealed that negative relative to neutral stimuli resulted in overall increased amplitudes in younger adults [*F*(1,25) = 9.46, *p* < 0.01, η_p_^2^ = 0.275] and this effect was reversed in older adults [*F*(1,25) = 14.60, *p* > 0.001, η_p_^2^ = 0.369].

##### P200 range

We found an interaction of congruence by age [*F*(1,50) = 7.45, *p* < 0.01, η_p_^2^ = 0.130]. Follow-up analysis revealed that congruent relative to incongruent stimuli elicited enhanced amplitude in younger adults [*F*(1,25) = 6.58, *p* < 0.02, η_p_^2^ = 0.208], but not in older adults [*F*(1,25) = 1.16, *p* > 0.2, η_p_^2^ = 0.045].

##### N200 range

The main effect of congruence was not significant [*F*(1,50) = 1.97, *p* > 0.1, η_p_^2^ = 0.038], but we observed an interaction of region by congruence [*F*(1,50) = 15.17, *p* < 0.001, η_p_^2^ = 0.233]. We resolved this interaction by region incongruent relative to congruent stimuli elicited increased amplitude in the posterior brain region [*F*(1,50) = 8.29, *p* < 0.01, η_p_^2^ = 0.249] and not anterior region [*F*(1, 50) = 1.24, *p* > 0.2, η_p_^2^ = 0.047].

We have further run a partial correlation (controlling for age) between the emotion-modulated N100 conflict effect [(negative incongruent - negative congruent) - (neutral incongruent - neutral congruent)] and the questionnaires data (i.e., ATQ, DASS). We found a significant negative correlation between the attentional shifting and the emotion modulated N100 conflict effect (*r* = -0.344, *p* = 0.007). This shows that lower levels of attentional shifting correlate with an increased benefit of negative emotions in the conflict sensitive N100. We also found a comparable correlation for effortful control (*r* = -0.259, *p* = 0.033), but this correlation did not survive correction for multiple comparisons. Finally, we also observed that the levels of stress (*r* = -0.308, *p* = 0.02) and anxiety (*r* = -0.302, *p* = 0.022) negatively correlated with the emotional N100 conflict effect, and this correlation was absent for the neutral emotional N100 conflict effect (all *p*s > 0.3). More specifically, lower levels of stress and anxiety correlated with a larger emotional N100 conflict effect but not the neutral conflict effect.

In summary, Experiment 1 tested the influence of *task-irrelevant* emotion on cognitive conflict processing in two age groups. We show that emotion facilitates RT conflict processing by reducing the conflict effect in both age groups. Further, negative emotion modulates cognitive conflict in the N100 and P200 time-ranges. Interestingly, older adults showed a valence-specific N100 conflict effect only at posterior electrode-site. Finally, emotion facilitated the N200 conflict effect in the older but not in younger adults, possibly reflecting prioritized processing of emotional information in older adults. To test an age-specific influence of the task relevance of emotion on emotional conflict processing we conducted Experiment 2.

## Experiment 2

### Methods

#### Participants

Participants who took part in Experiment 1 also participated in Experiment 2.

#### Stimulus Material and Procedure

We modified the original videos of Experiment 1 and created 12 congruent and 12 incongruent videos by matching or mismatching the emotional valence of the face and voice (e.g., voice pronouncing a neutral “A” and the corresponding audio “A” was pronounced emotionally, **Figure 1**). To create incongruent trials with a neutral target voice (i.e., audio trace), the original emotional facial video streams were combined with neutral auditory streams. Similarly, the originally neutral facial video streams were combined with emotional auditory streams to create incongruent trials with an emotional target. The original voice onset was used to align the incongruent voice with the lip movement in both incongruent conditions. The vocalization of the face and voice was always matched. Participants were instructed to report whether the voice was either emotional or neutral.

The duration of congruent and incongruent videos ranged between 1 and 2 s (**Table 4**). There were no differences between conditions in time before the audio onset and total video durations (see Supplementary Material for details).

**Table 4:** Timing of video stimuli of Experiment 2.

Video condition (the “vowel” specifies the interjection)	Time before start of the movement (ms)	Time before start of the audio stream (ms)	Total video duration (ms)
**Female**			
Neutralcongruent “A”	240	561	1400
Neutral congruent “O”	240	740	1480
Negative congruent “A”	240	665	1880
Negative congruent “O”	240	846	1840
Face neutral–voice negative “A”	240	590	1400
Face neutral–voice negative “O”	240	860	1480
Face negative–voice neutral “A”	240	683	1880
Face negative–voice neutral “O”	240	659	1840
**Male**			
Neutral congruent “A”	240	475	1400
Neutral congruent “O”	240	560	1400
Negative congruent “A”	240	450	1400
Negative congruent “O”	240	540	1400
Face neutral–voice negative “A”	240	328	1400
Face neutral–voice negative “O”	240	500	1400
Face negative–voice neutral “A”	240	590	1400
Face negative–voice neutral “O”	240	600	1400

Experiment 2 used a 2 (emotional, neutral) × 2 (congruent, incongruent) factorial design and consisted of four blocks. Each block comprised 52 videos (26 emotional and 26 neutral, half were congruent and the other half incongruent) presented in random order. To ensure that faces were not ignored, probe questions were introduced (10% of all trials), where participants had to report the emotional expression of the face. These trials were not used for further analysis (all participants answered >90% of questions correctly and were included for further analysis). Overall, 208 trials were presented and testing took approximately 45 min per participant.

#### EEG Recording and Pre-processing

The EEG recording and pre-processing were identical to Experiment 1. Artifact components were removed after ICA (mean = 16, SD = 3.6). Approximately 17% of the trials (incorrect responses, artifacts) were excluded from further analysis.

#### Data Analysis

Data analysis was identical to that of Experiment 1. In Experiment 2, a voice target (emotional or neutral) was preceded by either a congruent or an incongruent video. As we found motion differences for emotional compared to neutral videos (see Supplementary Material for details), a 200 ms baseline correction was applied before the voice onset to account for these differences.

### Results

#### Behavioral Data

##### RT data

The main effect of age was significant as older adults produced overall longer RTs than younger adults [see **Figure 2**; younger = 526 ms, SD = 66 ms; older = 740 ms, SD = 133 ms; *F*(1,50) = 63.918, *p* < 0.001, η_p_^2^ = 0.561]. Furthermore, we observer an interaction of emotion by age group [*F*(1,50) = 9.32, *p* < 0.01, η_p_^2^ = 0.157]. Emotional stimuli resulted in longer RTs in older adults [48 ms, SD = 10 ms; *F*(1,25) = 12.96, *p* < 0.01, η_p_^2^ = 0.341] but not in younger adults [-9 ms, SD = 8 ms; *F*(1,25) = 1.39, *p* > 0.2, η_p_^2^ = 0.053]. In a similar vein, the main effect of conflict varied as a function of age [interaction of congruence by age was significant, *F*(1,50) = 31.81, *p* < 0.01, η_p_^2^ = 0.389]: the main effect of congruence was larger in older adults [132 ms, SD = 11 ms; *F*(1,25) = 93.78, *p* < 0.01, η_p_^2^ = 0.790] than in younger adults [48 ms, SD = 6 ms; *F*(1,25) = 68.81, *p* < 0.01, η_p_^2^ = 0.734].

Finally, negative emotion influenced emotional control in an age-specific manner [emotion by congruence by age, *F*(1,50) = 13.79, *p* < 0.01, η_p_^2^ = 0.216]: the interaction of emotion by congruence was significant in older adults [*F*(1,25) = 17.95, *p* < 0.01, η_p_^2^ = 0.418] but not in younger adults [*F*(1,25) = 2.13, *p* = 0.157, η_p_^2^ = 0.078]. In older adults, the conflict effect was larger for neutral stimuli [170 ms, SD = 5 ms; *F*(1,25) = 160.25, *p* < 0.01, η_p_^2^ = 0.865] than for emotional stimuli [94 ms, SD = 17 ms; *F*(1,25) = 25.23, *p* < 0.01, η_p_^2^ = 0.502].

##### Error rate

We found a main effect of age as older adults made more errors than younger adults [*F*(1,50) = 4.14, *p* < 0.05, η_p_^2^ = 0.081]. The interaction of congruence by age was also significant [*F*(1,50) = 10.29, *p* < 0.01, η_p_^2^ = 0.179]: the main effect of congruence was larger in older adults [congruent = 1.5%, incongruent = 11.27%; *F*(1,25) = 21.49, *p* < 0.01, η_p_^2^ = 0.452] than in younger adults [congruent = 2.5%, incongruent = 4.37%; *F*(1,25) = 7.14, *p* < 0.02, η_p_^2^ = 0.254].

#### ERP Data

##### N100 range

**FIGURE 4:**
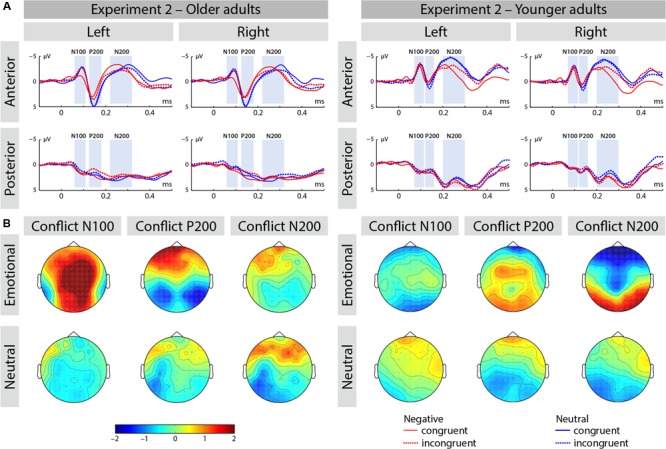
**(A)** ERP waveforms at selected averaged electrodes [left anterior (FP1, AF3, AF7, F3, F5, F7, FC3, FC5, FT7), right anterior (FP2, AF4, AF8, F4, F6, F8, FC4, FC6, FT8), left posterior (CP3, CP5, TP7, P3, P5, P7, PO3, PO7, O1), and right posterior (CP4, CP6, TP8, P4, P6, P8, PO4, PO8, O2)] depicting the conflict effect for emotional and neutral trials in Experiment 2. **(B)** Conflict effects are represented as a topographic distribution of amplitude differences (incongruent–congruent) for each of the ERP components (i.e., N100, P200, and N200).

As expected, the main effect of congruence was significant [**Figure 4**; *F*(1,50) = 12.86, *p* < 0.001, η_p_^2^ = 0.205], as well as an interaction of congruence by emotion [*F*(1,50) = 5.52, *p* < 0.05, η_p_^2^ = 0.099]. The conflict effect was larger in negative emotion stimuli [*F*(1,50) = 13.69, *p* < 0.001, η_p_^2^ = 0.215] relative to neutral stimuli [*F*(1,50) = 0.904, *p* > 0.3, η_p_^2^ = 0.018] and this effect was comparable across the both groups, as an interaction of emotion by congruence by age was not significant [*F*(1,50) = 0, *p* > 0.9, η_p_^2^ = 0].

##### P200 range

We found an interaction of region by congruence [*F*(1,50) = 8.84, *p* < 0.01, η_p_^2^ = 0.150]. Follow-up analysis revealed that congruent stimuli elicited increased amplitude relative to incongruent stimuli in the posterior brain region [*F*(1,50) = 5.32, *p* < 0.03, η_p_^2^ = 0.096], but not in the anterior brain region [*F*(1,50) = 0.165, *p* > 0.6, η_p_^2^ = 0.003]. This effect was comparable across both age groups, as an interaction of region by congruence by age was not significant [*F*(1,50) = 2.55, *p* > 0.1, η_p_^2^ = 0.049].

We also observed an interaction of region by emotion [*F*(1,50) = 15.31, *p* < 0.001, η_p_^2^ = 0.234], as well as an interaction of region by emotion by age [*F*(1,50) = 11.37, *p* < 0.001, η_p_^2^ = 0.185]. Follow-up analysis showed that an interaction of region by emotion was significant in older adults [*F*(1,25) = 19.76, *p* < 0.001, η_p_^2^ = 0.442], but not in younger adults [*F*(1,25) = 0.223, *p* > 0.6, η_p_^2^ = 0.009]. In older adults, emotional stimuli elicited reduced amplitude relative to neutral stimuli in the anterior brain region [*F*(1,25) = 16.81, *p* < 0.001, η_p_^2^ = 0.402], but not in the posterior brain region [*F*(1,25) = 1.61, *p* > 0.2, η_p_^2^ = 0.06].

##### N200 range

We observed an interaction of region by emotion [*F*(1,50) = 6.67, *p* < 0.02, η_p_^2^ = 0.118] and region by emotion by age [*F*(1,50) = 5.1, *p* < 0.03, η_p_^2^ = 0.093]. We resolved this interaction by age and found a significant two-way interaction of region by emotion in younger adults [*F*(1,25) = 13.72, *p* < 0.001, η_p_^2^ = 0.354], but not in older adults [*F*(1,25) = 0.046, *p* > 0.8, η_p_^2^ = 0.002]. In younger adults, neutral relative to emotional stimuli elicited increased amplitude in the anterior brain region [*F*(1,25) = 43.016, *p* < 0.001, η_p_^2^ = 0.632] and posterior region [*F*(1,25) = 13.39, *p* < 0.001, η_p_^2^ = 0.349].

Finally, we found an interaction of region by congruence [*F*(1,50) = 7.98, *p* < 0.01, η_p_^2^ = 0.138] and region by congruence by age [*F*(1,50) = 7.24, *p* < 0.01, η_p_^2^ = 0.127]. Further analysis showed an interaction of region by congruence in the older adults [*F*(1,25) = 24.39, *p* < 0.001, η_p_^2^ = 0.494], but not in younger adults [*F*(1,25) = 0.006, *p* > 0.9, η_p_^2^ = 0]. In older adults, congruent stimuli elicited increased amplitude relative to incongruent stimuli in the anterior brain region [*F*(1,25) = 4.36, *p* < 0.05, η_p_^2^ = 0.149], but not posterior brain region [*F*(1,25) = 1.14, *p* > 0.2, η_p_^2^ = 0.044].

With regard to the correlation analyses, in the emotional conflict task we only observed a positive correlation between activation control (i.e., the capacity to perform an action when there is a strong tendency to avoid it) and the emotionally modulated N100 conflict effect (*r* = 0.314, *p* = 0.012). That is, higher levels of activation control were associated with increased benefits of emotional stimuli in the N100 conflict effect.

In summary, Experiment 2 set out to test the role of emotionality of the target dimension in emotional conflict processing as a function of age. Consistent with our predictions, we show that emotional stimuli facilitated emotional conflict processing by reducing the RT conflict effect for older adults. Younger adults also showed a numerical trend in the same direction. The N100 responses showed an age-independent valence-specific reversal: incongruent negative stimuli elicited a larger amplitude than congruent stimuli, while neutral congruent trials elicited a larger amplitude than incongruent trials. Finally, we found an interaction of emotion and congruence in the P200 and N200 range across both age groups.

### Discussion

The present study tested the role of aging and the influence of emotion on cognitive and emotional conflict processing by using behavioral measures and ERPs of the EEG. In the following, we separately discuss the results of each conflict type and conclude with a general discussion.

#### Cognitive Conflict

In the cognitive conflict task, participants named the vowel expressed by the voice (i.e., “A” or “O”) irrespective of its emotional quality. Consequently, older adults produced overall longer RTs and took longer to respond to emotional than to neutral stimuli. Emotion sped up the response to conflict in both age groups. Finally, emotion modulated the N200 conflict effect in older adults only, while we observed valence-specific conflict-sensitive N100 and P200 responses in both age groups.

First, we were able to replicate previous findings and observed that negative emotion facilitate cognitive conflict processing by reducing the RT conflict effect (Kanske and Kotz, 2010, 2011a; Zinchenko et al., 2015). Emotional stimuli attract attention due to their motivational relevance for survival (Ohman et al., 2001; Vuilleumier et al., 2001) and trigger cognitive control processes (Norman and Shallice, 1986). Importantly, the magnitude of facilitation was similar in both younger and older adults. In other words, although older adults showed larger conflict effects and prolonged RTs to emotional stimuli, they preserved the emotional modulation of cognitive control (Dolcos et al., 2014).

In the EEG results we observed that the N100 conflict effect was larger for emotional than for neutral stimuli (Zinchenko et al., 2015). Importantly, we found this response pattern in both younger and older adults. In other words, negative emotion facilitates cognitive conflict processing as early as 100 ms after stimulus onset in both age groups, probably, by attracting additional attention to the target (Egner and Hirsch, 2005; Zinchenko et al., 2015). Interestingly, the conflict effect was absent in the N100 response to neutral stimuli of older but not younger adults (Hammerer et al., 2010). Conversely, the conflict-sensitive N100 was comparable across age groups when stimuli were emotional. A possible explanation could be that older adults benefit the most from processing of emotional compared to neutral stimuli (Scheibe and Carstensen, 2010). These results are also in line with findings that older rather than younger adults focus more on emotional information and that they can use emotional information during cognitive processing to compensate for their cognitive deficits (Scheibe and Blanchard-Fields, 2009; Mather, 2012; Mammarella et al., 2016). In other words, while negative emotion facilitates conflict processing in both age groups, older adults may be the primary beneficiaries in this process. In line with this idea, older adults also showed a reduced N100 for emotional relative to neutral trials, and this effect was reversed in younger adults. This shows that while emotional stimuli result in comparable neural and behavioral modulation of the conflict effect, the two groups may process emotional stimuli in an age-specific manner.

We also found a conflict effect in the P200 of younger but not older adults: incongruent P200 responses were smaller than congruent P200 responses. Increased allocation of attention has been linked to a decrease in the P200 amplitude (Crowley and Colrain, 2004; Kokinous et al., 2014). Therefore, we interpret the reduced P200 response in incongruent trials as an increase in attentional allocation to the target when it is incongruent (Etkin et al., 2006; Egner et al., 2008). Interestingly, while younger participants displayed an increased P200 response in incongruent trials, this effect was absent in older adults, that is, older adults showed no conflict effect in the P200 stimuli. Hammerer et al. (2010) reported that the magnitude of the conflict effect reduces with age, and older adults show a smaller ERP response in inhibition (also, see Kok, 2000 for a review). Therefore, the reduced ERP conflict effect goes along with an increased RT conflict cost.

Furthermore, we observed a conflict-related N200 response (Bruin and Wijers, 2002; Ho et al., 2015). Importantly, the N200 conflict effect was observed in both younger and older adults (Kokinous et al., 2014; Zinchenko et al., 2015). The N200 is evoked during tasks that require the inhibition of pre-potent responses, when two or more incompatible response tendencies are activated simultaneously (Van Veen and Carter, 2002; Nieuwenhuis et al., 2003). Therefore, the current results reflect conflict processing in both age groups.

To summarize, although older adults showed an overall decline in cognitive control and prolonged responses to emotional stimuli, the influence of emotion on cognitive conflict processing was preserved in the elderly. Importantly, the neural correlates of affective facilitation of cognitive control point to the particular benefit for the elderly from processing emotional information.

#### Emotional Conflict

In the emotional conflict task, participants reported the emotional valence of a presented vowel irrespective of whether the emotion expressed in the face was congruent or not, while the lip movement (face) and vocalization (voice) were congruent and task-irrelevant. As a result, older adults responded more slowly and displayed larger conflict effects than younger adults. Further, the behavioral results reveal that emotion facilitates conflict processing but only in older participants. The N100 response showed a valence-specific conflict effect in both age groups: incongruent stimuli elicited larger N100 amplitude than congruent stimuli, and this effect was greater in negative relative to neutral stimuli. Furthermore, we found conflict- and valence-specific effects in the P200 and N200 responses.

Interestingly, emotional stimuli facilitated RT conflict processing in older adults, while younger adults only showed a numerical trend toward significance. A previous study showed that negative emotion targets facilitate *emotional conflict* processing (Zinchenko et al., 2015). Processing of emotional stimuli enhances the readiness to act (Oatley and Jenkins, 1996) and speeds up executive control not only in cognitive conflict but also in emotional conflict processing (Kanske, 2012; Zinchenko et al., 2015). Importantly, our findings confirm that the elderly preserve the beneficial role of negative emotion in the emotional conflict processing.

Contrary to the results of Samanez-Larkin et al. (2009), we observed a large conflict effect in emotional incongruence in both younger and older adults. In other words, older adults showed no advantage over younger adults in emotional conflict processing. One of the possible explanations for this is that the current study used dynamic multisensory stimuli, which made the task very demanding and resulted in larger conflict for older compared to younger adults. Indeed, Samanez-Larkin et al. (2009) observed no difference in the error rates between younger and older adults, while in our task this difference was substantial. Previous studies showed that older adults particularly benefit from processing of multisensory stimuli (Laurienti et al., 2006; Peiffer et al., 2007; Diederich et al., 2008; Hugenschmidt et al., 2009; DeLoss et al., 2013). However, our findings imply that incongruent multisensory stimuli may, in turn, be especially difficult to process for older adults.

Furthermore, we found valence-specific conflict effect in the N100: incongruent stimuli elicited a larger N100 response to incongruent trials in the negative emotion condition. Importantly, this effect was absent in the neutral condition. Zinchenko et al. (2015) suggested that the valence-specific dissociation in the N100 reflects a conflict-specific processing mechanism. In more detail, unlike cognitive conflict, *emotional* conflict processing has been found to involve facilitated *inhibition of the task-irrelevant* emotional distractor dimension of a stimulus (Etkin et al., 2006; Egner et al., 2008). Furthermore, studies showed that the inhibition of neutral task-irrelevant distractors is less demanding than to emotional distractors (Ohman et al., 2001; Vuilleumier et al., 2001). Therefore, we assume that the observed N100 conflict effect for emotional rather than neutral stimuli may reflect a valence-specific mechanism of inhibition effective in emotional conflict processing.

In line with our previous findings, incongruent stimuli produced reduced P200 at posterior but not anterior electrode-sites (Zinchenko et al., 2015). A decreased P200 response has been linked to an increase in the allocation of attention (Crowley and Colrain, 2004). Therefore, a reduced P200 response in incongruent trials could imply attentional capture by mismatching vocalizations. As incongruent stimuli require more resources to be inhibited than neutral stimuli (Algom et al., 2004; Dolcos and McCarthy, 2006), we interpret the observed results as less allocation of attention to the vocalization when the distractor was incongruent rather than congruent. Importantly, we show that this effect holds true for older adults as well.

Finally, we also report a conflict-sensitive N200 (Bruin and Wijers, 2002; Kokinous et al., 2014; Ho et al., 2015; Zinchenko et al., 2015). Interestingly, this conflict effect appeared in older but not in younger adults. Moreover, unlike in the cognitive conflict task, incongruent stimuli resulted in a reduced N200 amplitude in older adults. Previous findings suggested that the N200 indexes conflict monitoring and its amplitude reflects the extent to which attentional control is required for conflict processing (Van Veen and Carter, 2002; Nieuwenhuis et al., 2003; Dennis and Chen, 2007). Therefore, a reduced N200 in response to incongruent stimuli may reflect reduced effortful control and the impeded ability to engage executive processes in older relative to younger adults (Posner and Rothbart, 2007). This is in line with the increased number of incorrect responses to incongruent stimuli for older relative to younger adults.

## General Discussion and Limitations

In contrast to substantial cognitive and physical decline, older adults seem to maintain high levels of affective well-being and emotional stability (Scheibe and Carstensen, 2010). Our data show that negative emotions can effectively modulate cognitive and emotional control in healthy aging, when using multisensory real-life stimuli. An important implication of the current findings is that healthy older adults preserve the ability to process conflicting emotional information (e.g., in irony; Wang et al., 2007; Pexman, 2008; Watanabe et al., 2014; Zinchenko et al., 2015). In this regard, it was shown that the correct recognition of emotional states facilitates social behavior and interpersonal communication (Shimokawa et al., 2001; Blair, 2005; Chaby et al., 2015). Therefore, the present results provide an important contribution to understanding of changes in emotional competencies in healthy aging (Doerwald et al., 2016).

Although emotion facilitated both cognitive and emotional conflict processing in older adults, the neural correlates of conflict processing were different. More specifically, in the cognitive conflict task we observed the main effect of congruence in the N200 over the posterior electrode-sites, while in the emotional conflict task the conflict-sensitive N200 was found at anterior electrode-sites. Various theories attempted to describe age-related decline in executive functions. The frontal aging theory postulates that reduced conflict-sensitive ERP activity over frontal electrode-sites may arise from structural and functional deterioration of the frontal lobes (Lucci et al., 2013). On the other hand, the age-related posterior–anterior shift theory argues that age-related increased frontal lobe activity is a consequence of decreased activity in posterior brain regions (Davis et al., 2008; St. Jacques et al., 2009). Our findings indirectly support both assumptions that vary as a function of how a task engages conflict-specific mechanisms (Egner and Hirsch, 2005; Etkin et al., 2006). In particular, cognitive conflict processing (Experiment 1) amplifies the processing of the stimulus target dimension (Egner and Hirsch, 2005), while emotional conflict processing (Experiment 2) seems to lead to facilitated inhibition of the non-target emotional distractor dimension of a stimulus (Etkin et al., 2006). Therefore, our findings add important evidence to the literature that aging does not affect conflict processes equally but rather shows conflict-specific modulations of interference control (Kawai et al., 2012; Sebastian et al., 2013). These results are particularly compelling as we used the same multisensory stimuli in the two different conflict tasks.

In cognitive conflict processing, task-irrelevant emotion prolonged RTs in both age groups. It has been shown that salient emotional stimuli capture attention and need longer to be processed (Compton, 2003; Vuilleumier, 2005). These findings are consistent with previous study that tested the role of negative emotion in cognitive conflict processing using the same multisensory audio-visual stimuli (Zinchenko et al., 2015), but not consistent with previous studies that used unimodal audio (Kanske and Kotz, 2011a) and visual stimuli (Kanske and Kotz, 2011b) and did not observe overall increased responses for emotional stimuli. Possibly, multisensory dynamic stimuli elicit stronger emotional responses and hinder the RTs relative to unimodal stimuli. Importantly, negative emotion has consistently been show to facilitate cognitive control and reduce the conflict effect (i.e., incongruent–congruent) irrespective of stimulus type. On the other hand, older, but not younger adults showed prolonged responses to negative relative to neutral stimuli in emotional conflict task. Older adults prioritize processing of emotional information and preserve the ability to process and regulate emotions, despite a general decline in executive control (Charles and Carstensen, 2007; St. Jacques et al., 2010). Possibly, the preserved ability to process emotional information in healthy aging happens at the expense of prolonged responses. Additionally, task-relevance may modulate the influence of negative emotion on the speed of responses in older adults and may possibly cause the observed differences across conflict types (Lichtenstein-Vidne et al., 2012). Therefore, the influence of negative emotion on response duration may potentially depend on the stimulus characteristics (unimodal, multisensory) and task relevance (task-relevant, -irrelevant), and future studies will need to address these points more systematically.

People of different ages are more likely to attend to and report more exposure to faces of their own than another age group (Anastasi and Rhodes, 2005; Lamont et al., 2005; Ebner and Johnson, 2010; Ebner et al., 2012). However, in the present study we only used videos of younger individuals, which could limit the observed effects as older adults may have processed faces of younger adults differently relative to their own-age faces (He et al., 2011). This factor should be further considered in future studies. In line with previous findings (Kanske and Kotz, 2012), our current results indicated that participants’ interpersonal characteristics modulate the role of emotions on cognitive and emotional conflict processing. We show that individual abilities to initiate and shift attention, as well as general levels of stress and anxiety modulate the role of emotion in conflict processing (Kanske and Kotz, 2012). Therefore, future studies should also consider inter-individual parameters when examining emotions and executive control. Lastly, as the current study concentrated on negative emotions, one question that remains is whether we would observe the same result in positive emotional conflicts in older participants as in younger adults (Zinchenko et al., 2017). For instance, while some previous studies reported a positivity effect: a trend in older adults to preferentially orient their attention to and remember more positive than negative or neutral stimuli (Kennedy et al., 2004; Mather and Carstensen, 2005; Sasse et al., 2014), other studies have failed to replicate such a positivity effect in older adults (Murphy and Isaacowitz, 2008; Steinmetz et al., 2010). Since previous studies showed that older adults benefit from processing of multisensory information (Hugenschmidt et al., 2009; DeLoss et al., 2013), we hypothesize that salient multimodal real-life stimuli (see Zinchenko et al., 2017) may clarify the role of positivity in processing of emotional information (Reed and Carstensen, 2012). It was proposed that the effect of positivity might operate during an active regulation of emotional states (Isaacowitz and Blanchard-Fields, 2012). Therefore, it is possible that the positivity effect can be observed when emotion is task-relevant (in an emotional conflict task), relative to a cognitive conflict task, when emotion is task-irrelevant. More specifically, it is plausible that older adults may show no cost of processing of neutral stimuli in the presence of a positive emotion distractor (Zinchenko et al., 2017). Alternatively, a meta-analysis by Reed et al. (2014) showed that positivity effect is larger in studies that do not constrain cognitive processing. As both cognitive and emotional conflict tasks tax executive functions, it is plausible that the age-specific positivity effect may be absent in these two tasks. Therefore, the question remains whether positive emotions influence cognitive and emotional control differently as a function of age. Future studies should address these questions in more detail.

## Conclusion

While aging brings apparent changes to cognitive functions such as executive control, memory, attention, and processing speed, it seems to have little effect on the way processing of task-relevant and -irrelevant emotional information influences the two investigated control systems. The influence of emotion on cognitive and emotional conflict processing remains intact in age, possibly due to age-related changes in motivation and/or differential rates of brain deterioration across neural systems.

## Author Contributions

AZ and SK conceived and designed the experiments. AZ performed the experiments. AZ, CO, and PK analyzed the data. AZ, SK, CO, PK, ES, and AV interpreted the results. AZ wrote first draft of paper. SK, CO, PK, ES, and AV revised the paper. AV accessed participants.

## Conflict of Interest Statement

The authors declare that the research was conducted in the absence of any commercial or financial relationships that could be construed as a potential conflict of interest.
