# Supplementary material for: The Influence of Negative Emotion on Cognitive and Emotional Control Remains Intact in Aging
Source: Front Aging Neurosci. 2017 Nov 1;9:349. doi: 10.3389/fnagi.2017.00349 (PMC5671981; doi:10.3389/fnagi.2017.00349)
Supplement: Supplementary file 1 [file Data_Sheet_1.docx]

Supplemental Materials

**Experiment 1**

**Timing**

There was no significant delay difference between the start of the video and the voice onset of videos between vocalizations (i.e., “A” and “O”, paired-t(7) = -1.98, *p* > 0.09, *d* = -0.115), emotions (i.e., neutral and emotional, paired-t(7) = -1.24, *p* > 0.26, *d* = -0.072), and congruence (i.e., congruent, incongruent, paired-t(7) = 0.01, *p* > 0.4, *d* = 0.001) (see Table 1). For the total video duration, there was no significant difference between vocalizations (paired-t(7) = -1.86, *p* > 0.1, *d* = -0.067) and congruence (paired-t(7) = 0.58, *p* > 0.5, *d* = 0.021). The total video duration was longer for negative than neutral videos (paired-t(7) = -3.22, *p* < 0.05, *d* = -0.116).

**Experiment 2**

Timing

There was no significant delay between the start of the video and the voice onset of videos between vocalizations (i.e., “A” and “O”, paired-t(7) = 1.49, *p* > 0.1, *d* = 0.855), emotions (i.e., neutral and emotional, paired-t(7) = 0.211, *p* > 0.8, *d* = 0.012), and congruence (i.e., congruent, incongruent, paired-t(7) = 0.420, *p* > 0.6, *d* = 0.001) (see Table 3). For the total video duration, there was no significant difference between vocalizations (paired-t(7) = 1.54, *p* > 0.1, *d* = 0.055), congruence (paired-t(7) = 0.0, *p* = 1) and emotions (paired-t(7) = 0.0, *p* = 1).

**Ratings of older adults**

Complete videos, video streams alone, and audio streams alone were rated on a 7-point Likert scale using Self-Assessment Manikins for expressiveness, arousal, and emotion identification (Bradley & Lang, 1994) by 26 (14 female) older adults (see Table 2). A paired-samples t-test for the complete videos showed no main effect of emotion for either arousal (paired-t(25) = -0.908, *p > 0*.3, *d* = -0.363) or for expressiveness (paired-t(25) = -0.496, *p >* 0.6, *d* = -0.198). The main effect of emotion was significant in the ratings of valence: participants rated emotional videos as more emotional than neutral videos (paired-t(25) = 23.789, *p* < 0.01, *d* = 9.515). For the audio streams alone, the result of the analysis showed no main effect of emotion for either arousal (paired-t(25) = 0.739, *p >* 0.4, *d* = 0.296) or for expressiveness (paired-t(25) = -0.372, *p >* 0.7, *d* = -0.149). However, the main effect of emotion was significant for the ratings of valence (paired-t(25) = 25.563, *p <* 0.01, *d* = 10.225): emotional videos were rated as more emotional than neutral videos. For the video stream alone, there was no main effect of emotion for either arousal (paired-t(25) = -1.279, *p >* 0.2, *d* = 0.512) or for expressiveness (paired-t(25) = -1.137, *p >* 0.2, *d* = -0.455). The main effect of valence was significant (paired-t(25) = 10.912, *p <* 0.001, *d* = 4.365).

**Ratings of younger adults**

A paired-samples t-test for the complete videos showed no main effect of emotion for either arousal (paired-t(25) = 0.485, *p > 0*.6, *d* = 0.194) or expressiveness (paired-t(25) = -0.658, *p >* 0.5, *d* = -0.263). The main effect of emotion was significant in the ratings of valence: participants rated emotional videos as more emotional than neutral videos (paired-t(25) = 18.402, *p* < 7.361). For the audio streams alone, the result of the analysis showed no main effect of emotion for either arousal (paired-t(25) = -1.232, *p >* 0.2, *d* = -0.493) or expressiveness (paired-t(25) = -1.081, *p >* 0.2, *d* = -0.432). However, the main effect of emotion was significant for the ratings of valence (paired-t(25) = 34.119, *p <* 0.001, *d* = 13.648): emotional videos were rated as more emotional than neutral videos. For the video stream alone, there was no main effect of emotion for either arousal (paired-t(25) = 0.658, *p >* 0.5, *d* = 0.263) or for expressiveness (paired-t(25) = 0.485, *p >* 0.6, *d* = 0.194). The main effect of valence was significant (paired-t(25) = 18.402, *p <* 0.001, *d* = 7.361).

*Habitation effect*

To test for a potential habituation effect to emotional stimuli (visual and auditory), we have ran an additional 4 x 2 x 2 rm ANOVA with factors blocks (1-4) x congruence (congruent, incongruent) x emotion (negative, neutral) and age as a between group factor separately for Experiments 1 and 2. In the cognitive conflict Experiment 1, we found the main effect of block (F(3, 150) = 9.084, p < 0.001, η_p_^2^ = 0.154). This effect was caused by overall reduced RTs in block 1 (600 ms) relative to block 4 (569 ms, F(1, 51) = 14.15, p < 0.001, η_p_^2^ = 0.217), as participants’ performance improved with more practice. However, none of the interactions of interest were significant: block x congruence x emotion (F(3, 150) = 1.76, p > 0.157, η_p_^2^ = 0.034), block x congruence x emotion x age (F(3, 150) = 0.649, p > 0.5, η_p_^2^ = 0.013).

In the emotional conflict Experiment 2, we again found the main effect of block (F(3, 150) = 9.66, p < 0.001, η_p_^2^ = 0.162). With practice, participants’ responses got overall faster in block 4 (631 ms) relative to block 1 (673 ms; F(1, 51) = 14.92, p < 0.01, η_p_^2^ = 0.226). Finally, we found no interactions of blocks with factors of interest: block x emotion x congruence (F(3, 150) = 1.95, p > 0.124, η_p_^2^ = 0.038) and block x emotion x congruence x age (F(3, 150) = 1.28, p > 0.28, η_p_^2^ = 0.025).

To summarize, although participants’ RTs were speeded generally as a function of block, we found no evidence of saturation effect.
